# Chondroid syringoma: case series

**DOI:** 10.1093/jscr/rjaf187

**Published:** 2025-04-09

**Authors:** Imane Boujguenna, Sara Badja, Mounir Jaafari, Soufiane Abdouh, Fatima Boukis

**Affiliations:** Guelmim Faculty of Medicine and Pharmacy, Ibn Zohr Agadir University, Guelmim 81000, Morocco; Department of Otolaryngology, Head and Neck Surgery, Guelmin Regional Hospital, Guelmim 81000, Morocco; Faculty of Medicine and Pharmacy of Guelmim, Ibnou Zohr University Agadir, Guelmim 81000, Morocco; Marrakesh Faculty of Medicine and Pharmacy, Cadi Ayyad University, Marrakesh 4000, Morocco; Pathological Anatomy Laboratory, Guelmim 81000, Morocco

**Keywords:** chondroid syringoma, histopathology, literature review

## Abstract

Chondroid syringoma (CS) is a rare benign cutaneous neoplasm originating from the exocrine glands, representing ⁓0.01% of all primary skin cancers. This report presents three cases of CS: one located at the infraorbital rim and two in the cheek region. The diagnosis in all cases was confirmed through histopathological examination. Treatment involved surgical excision with multidisciplinary collaboration to ensure optimal patient care. These findings emphasize the importance of accurate histopathological diagnosis and coordinated specialist efforts for effective management of this uncommon tumor.

## Introduction

Chondroid syringoma (CS) is a rare benign cutaneous neoplasm of the exocrine glands, representing only 0.01% of all primary skin cancers [1]. These tumors most commonly occur in the head and neck region. They typically present between the ages of 20 and 60 and are more frequent in men than in women. We report three cases of CSs: the first located at the infraorbital rim, and the second and third in the cheek region.

## Case presentations

### Case 1

**Figure 1:**
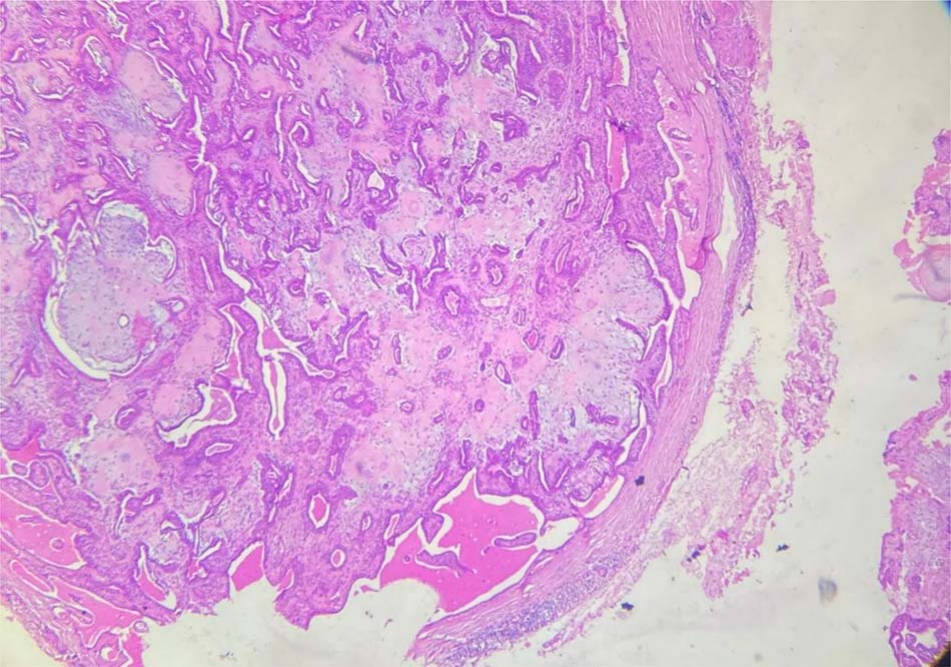
Well-circumscribed benign tumor proliferation.

A 48-year-old Moroccan woman, with no significant medical history, presented with a nodule on the infraorbital rim evolving over 1 year without associated symptoms. Clinical examination revealed a 0.7 cm nodular swelling at the infraorbital rim below the lower eyelid. It was mobile and skin-colored, without inflammatory signs. Ophthalmologic examination was unremarkable. Lymph nodes and salivary glands were normal. An excisional biopsy of the mass was performed. Macroscopically, it was a 0.7 cm beige, firm nodule. Microscopic examination showed a well-circumscribed benign tumor proliferation (Fig. 1) composed of tubules, ducts, and cysts lined by a bilayered epithelium. The cells exhibited no nuclear atypia. The stroma was fibromyxoid with chondroid foci. There was no perineural invasion or vascular emboli, and surgical margins were clear. Given this typical microscopic appearance, no additional studies were indicated, and the final diagnosis was CS. Postoperative recovery was uneventful, and no further follow-up or additional examinations were necessary.

### Case 2

**Figure 2:**
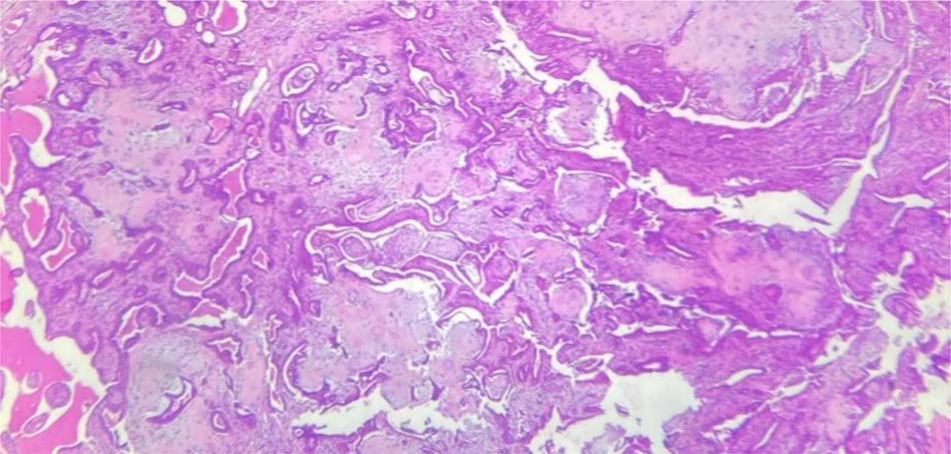
Benign tumor proliferation composed of cysts, tubules, and ducts.

**Figure 3:**
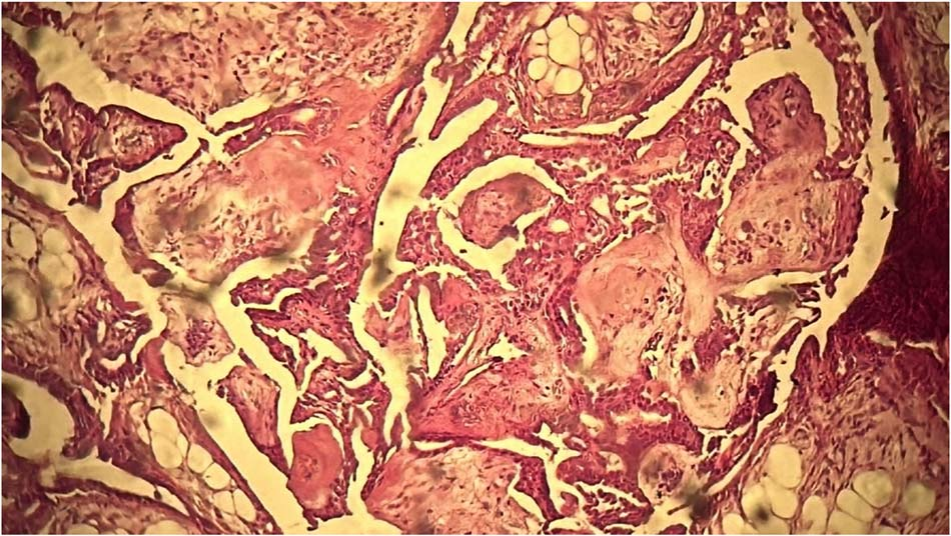
The cells exhibited no nuclear atypia.

A 53-year-old Moroccan man, with no significant medical history, presented with a cheek swelling evolving over 6 months without associated symptoms. Clinical examination revealed a 1.7 cm cystic-like swelling in the cheek, suggestive of a ruptured epidermoid cyst. It was mobile and skin-colored, without inflammatory signs. Lymph nodes and salivary glands were normal. An excisional biopsy of the mass was performed. Macroscopically, it was a 1.7 cm beige, friable to firm, rounded nodule. Microscopic examination showed a well-circumscribed benign tumor proliferation composed of cysts, tubules, and ducts (Fig. 2), lined by a bilayered epithelium. The cells exhibited no nuclear atypia (Fig. 3). The fibromyxoid and chondroid stroma allowed for diagnosis without additional techniques. There was no perineural invasion or vascular emboli, and surgical margins were clear. Postoperative recovery was uneventful, and no further follow-up or additional examinations were necessary.

### Case 3

**Figure 4:**
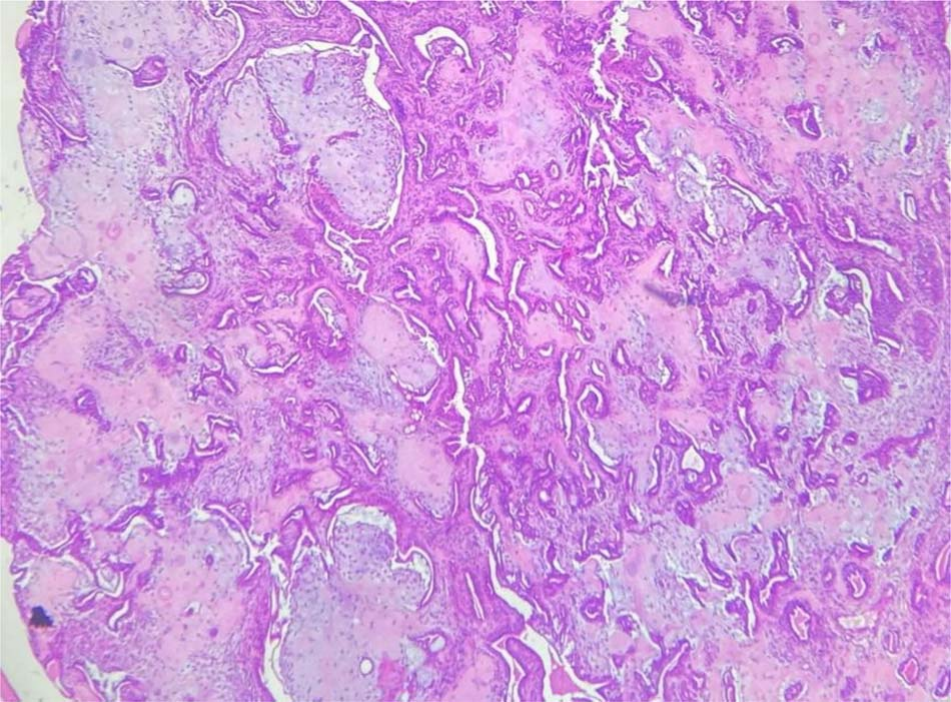
Chondroid stroma.

A 62-year-old Moroccan man, with no significant medical history, presented with a cheek swelling evolving over 9 months without associated symptoms. Clinical examination revealed a 1.2 cm nodular swelling in the cheek. It was mobile and skin-colored, without inflammatory signs. Lymph nodes and salivary glands were normal. An excisional biopsy of the mass was performed. Macroscopically, it was a 1.2 cm beige, friable to firm, rounded nodule. Microscopic examination showed a well-circumscribed benign tumor proliferation composed of cysts, tubules, and ducts, lined by a bilayered epithelium. The cells exhibited no nuclear atypia. The fibromyxoid and chondroid stroma (Fig. 4) allowed for diagnosis without additional techniques. There was no perineural invasion or vascular emboli, and surgical margins were clear. Postoperative recovery was uneventful, and no further follow-up or additional examinations were necessary.

## Discussion

CS was first described by Hirsch and Helwig in 1961 [36]. It is a benign tumor with rare recurrences; however, in rare cases of malignant CSs, the metastasis rate is 60%, and the mortality rate is 25% [25]. This tumor originates from eccrine or apocrine sweat glands. It is very rare, accounting for only 0.01% of all primary cutaneous neoplasms [37]. It presents as a solitary, slow-growing, painless intradermal or subcutaneous nodule, predominantly affecting the head and neck region [38]. We report all cases cited in the literature of CSs of the cheek (Table 1) and orbital region (Table 2). It more commonly affects middle-aged men. Tumor size can vary from 2 mm to over 1 cm. Clinically, differential diagnoses include neurofibroma, dermoid cysts, sebaceous cysts, dermatofibroma, lipoma, pilomatrixoma, histiocytoma, seborrheic keratosis, or basal cell carcinoma. Hirsch and Helwig [36] proposed five histological criteria for its diagnosis: (i) nests of cuboidal or polygonal cells; (ii) intercommunicating alveolar tubular structures lined by two or more rows of cuboidal cells; (iii) ductal structures composed of one or two rows of cuboidal cells; (iv) occasional cysts with keratin; (v) a variably composed matrix. Features suggesting malignant transformation include severe atypia, high cellularity, high mitotic index, infiltration of surrounding tissues, and tumor necrosis. Immunohistochemistry is generally not necessary for diagnosis. The inner epithelial layer shows expression of the following antibodies: keratin, EMA, CEA, GCDFP-15, actin, cytokeratin 15; and the myoepithelial layer shows expression of antibodies: S100, SOX10, NSE, GFAP, SMA, calponin, p63, with nuclear expression of PLAG1. CSs are rare tumors. The histological differential diagnosis includes cutaneous chondromas and myoepitheliomas, which lack epithelial structures, and papillary hidradenomas, which do not exhibit a chondroid stroma [39]. The gold standard treatment is surgical excision, aiming to preserve the patient’s esthetic appearance, especially given the tumor’s predominant cephalic location [31]. The likelihood of postoperative recurrence is very low, indicating that follow-up is not necessary in benign cases [40]. CSs are relatively rare tumors. Diagnosis is histopathological. Optimal management relies on effective collaboration among the various healthcare professionals involved.

**Table 1:** Cases of benign CSs of the cheek.

**Case**	**Year**	**Country**	**Gender**	**Age**	**Treatment**
[1]	2022	Pakistan	M	45	Surgical excision
[2]	2021	India	F	42	Surgical excision
[3]	2021	Ghana	M	33	Surgical excision
[4]	2020	Italy	F	70	Nd:Yag laser
[5]	2019	India	F		Surgical excision
[6]	2017	Korea	F	46	Surgical excision
[7]	2014	India		Child	Surgical excision
[8]	2014	India	F	42	Surgical excision
[9]	2013	India	M	40	Surgical excision
[10]	2011	India	M F	16 21	Surgical excision
[11]	2009	India	M		Surgical excision
[12]	2009	Syria	F	17	Surgical excision
[13]	2007	USA	M		Surgical excision
[14]	2004	UK	M	40	Surgical excision
[15]	1996	Japan	M	35	Surgical excision
[16]	1987	UK			Surgical excision

**Table 2:** Cases of benign orbital CSs.

**Case**	**Year**	**Country**	**Age**	**Gender**	**Treatment**
[17]	1993	Italy	81	M	Excisional biopsy
[18]	1999	Japan			Excisional biopsy
[19]	2006	Turkey	46	M	Excisional biopsy
[20]	2006	Algeria	62	F	Surgical resection
[21]	2006	India	45	M	Excisional biopsy
[22]	2007	USA	Age from 18 to 64		
[23]	2007	Swiss	84	M	
[24]	2011	India	56	M	Surgical resection
[25]	2012	Morocco	41	F	Left lateral orbitotomy + surgical resection
[26]	2013	USA			
[27]	2014	Greece	53	M	Excisional biopsy
[28]	2018	India	60	M	Enucleation
[29]	2019	USA	19	F	Excisional biopsy
[30]	2019	Morocco	45	M	Excisional biopsy
[31]	2019	India	60	M	Surgical resection
[32]	2022	Niger	35	M	Surgical resection
[33]	2023	India	58	M	Exenteration surgery of the right eye + postoperative radiation therapy
[34]	2024	India	18	M	Excision of the mass
[35]	2024	India	55	M	Excision of the mass
